# The prediction of nursing students’ knowledge and self-efficacy in pediatric pain management

**DOI:** 10.1186/s12912-025-04119-0

**Published:** 2025-12-24

**Authors:** Bahise Aydın, İlknur Bektaş, Murat Bektaş

**Affiliations:** 1 Department of Nursing, Faculty of Health Sciences, Izmir University of Economics, Sakarya Street No:156, 35330 Balçova, Izmir, Türkiye; 2 Department of Nursing, Faculty of Health Science, İzmir Bakırçay University, İzmir, Türkiye; 3 Faculty of Nursing, Dokuz Eylül University, İzmir, Türkiye

**Keywords:** Nursing, Student, Pain management, Knowledge, Self-efficacy

## Abstract

**Background:**

Pediatric pain is a complex and often undertreated issue. Nurses’ knowledge and self-efficacy in pediatric pain management are essential to improving pain outcomes. Undergraduate nursing education plays a critical role in developing these competencies, yet the relationship between knowledge and self-efficacy in this area remains underexplored.

**Purpose:**

This study aimed to predict the pediatric pain management knowledge levels of nursing students based on their self-efficacy.

**Methods:**

This descriptive and correlational study included 227 third- and fourth-year nursing students enrolled in the Child Health and Disease Nursing course at a state university in Türkiye. Data were collected using a sociodemographic data form, the Pediatric Pain Management Knowledge Scale, and the Pediatric Pain Management Self-Efficacy Scale. Descriptive statistics, correlation, and linear regression analysis were performed.

**Results:**

Participants’ mean age was 21.40 ± 1.38 years; 59% were female, and 51.1% had prior education on pain management. Knowledge scores for “Barriers”, “Pain Diagnosis,” and “Pain Assessment” significantly predicted the “Decision-Making” subdimension of the self-efficacy scale. Additionally, the “Pain Pathophysiology” subdimension significantly predicted the “Planning and Management” subdimension scores (*p* < 0.05). The knowledge subdimensions explained 87.4% of the variance in “Decision-Making” and 14.8% in “Planning and Management”.

**Conclusions:**

Nursing students’ overall knowledge levels of pediatric pain management did not predict their self-efficacy. However, a strong and statistically significant relationship was found between the subdimensions of “Pain Awareness,” “Pathophysiology,” “Barriers,” “Assessment,” and “Pain Control” knowledge and the “Decision-Making” subdimension of self-efficacy. These findings highlight the importance of targeted education on pediatric pain content to strengthen students’ self-efficacy in clinical practice.

**Trial registration:**

Not applicable.

## Introduction

Pain is frequently encountered in childhood and can adversely impact children’s developmental milestones, emotional well-being, and recuperation. Pain not adequately managed can have negative physiological, psychosocial, and economic consequences for children, their families, and society [1]. Pain is a symptom that arises in children due to illness, trauma, or medical procedures and requires effective management [2]. Effective pediatric pain management is essential because unrelieved pain adversely affects children’s recovery, daily functioning, and family well-being; timely and adequate control improves satisfaction, shortens hospitalization, and reduces healthcare costs [1, 2].

Nurses are the most crucial healthcare team members in pediatric pain management. However, they are also frequently the primary practitioners of painful procedures due to various medical interventions and care practices, such as vaccination, medication administration, and blood sampling [1–3]. This necessitates that nurses possess the knowledge and skills to manage the child’s pain and procedural pain. Nurses’ knowledge levels, behaviors, and self-efficacy in pain management significantly affect nursing care [2, 3].

Recognizing, diagnosing, and assessing pain in children are among the most challenging aspects for healthcare professionals. Studies in the literature indicate that nurses working with pediatric patients experience various difficulties and inadequacies in this regard [4–6]. Equipping nursing students with a comprehensive foundation in pain assessment and management before entering clinical roles is imperative [7, 8]. However, in the literature, studies evaluating the knowledge and attitudes of student nurses enrolled in pediatric nursing courses regarding children’s pain have revealed that students have a lack of knowledge about pain assessment and pharmacological and non-pharmacological pain management [9, 10].

Effective pediatric pain management requires not only theoretical knowledge but also strong self-efficacy, which enables nurses to confidently perform and maintain clinical skills. Knowledge alone may not guarantee the consistent implementation of appropriate interventions. Nurses’ ability to identify required information, choose age- and condition-appropriate strategies, and evaluate patient outcomes is closely linked to their self-efficacy level [10, 11]. Effective pediatric pain management requires nurses to apply age-appropriate assessment methods, personalize interventions based on the child’s needs, and monitor outcomes to ensure optimal care [12].

The literature indicates that nurses with high self-efficacy possess strong knowledge application and decision-making skills and establish better collaboration with parents and team members regarding pediatric pain management [12]. Therefore, in addition to the knowledge and skills of pediatric nurses in pain management, their self-efficacy should also be assessed. Numerous studies in the literature have examined nurses’ knowledge, attitudes, and behaviors regarding pediatric pain management. However, few studies have specifically explored how nursing students’ pain-related knowledge correlates with their self-efficacy [13, 14]. Determining the pediatric pain knowledge levels and self-efficacy in pain management of nursing students may also enable the evaluation and development of the current curriculum. For this reason, this study was designed to predict nursing students’ knowledge levels and self-efficacy regarding pediatric pain management.

**Research Question**:

• To what extent do nursing students’ pediatric pain management knowledge levels predict their self-efficacy in pain management in children?

## Methods

### Study design

A cross-sectional design employing descriptive and correlational methods was utilized in this study.

### Study setting and sample size

Participants included third-year students taking the pediatric nursing course and fourth-year students engaged in clinical pediatric internships at a public university in Türkiye. Data collection was conducted throughout the fall and spring terms of the 2021–2022 academic year.

The sample size of the study was determined using the G*Power 3.1.9.7 program for a linear multiple regression analysis. The effect size used in the calculation was based on the findings of the study conducted by Aydın and Bektaş [9]. According to the power analysis, with an effect size of 0.292 (f² = 0.292), a significance level of 0.01 (α = 0.01), and a power of 0.99 (1-β = 0.99), the minimum required sample size was calculated as 184. To account for possible data loss or incomplete responses, the sample size was increased by 20%, resulting in a target of 221 participants [15, 16].

However, in order to enhance the representativeness of the sample and minimize nonresponse bias, all 300 eligible students (240 third year and 60 fourth year) enrolled at the state university were invited to participate in the study. Ultimately, data were collected from 227 students who voluntarily agreed to participate. Including a larger sample than the minimum required strengthened the statistical power of the study, increased the precision of the estimates, and enabled subgroup analyses. The results of three regression analyses were used for post-hoc power in this study. The analysis determined that the power of the study ranged from 0.83 to 0.99.

### Data collection

Data were gathered via three instruments: a demographic information form, a scale assessing nursing students’ pediatric pain knowledge, and a self-efficacy scale designed for evaluating pediatric pain management capabilities.

#### Student information form

The researchers developed this form, which consists of seven questions regarding students’ age, gender, grade point average, type of high school graduation, previous pediatric pain training, and, if applicable, the type of training received.

#### Pediatric pain management knowledge scale for nursing students

This scale was developed by Aydın and Bektaş [17] to assess student nurses’ pediatric pain management knowledge. The scale consists of six sub-dimensions and a total of 29 items to evaluate students’ knowledge levels regarding pain awareness, pain pathophysiology, barriers to pain management, pain diagnosis, assessment, and control. Responses were rated on a five-point Likert scale ranging from 1 (strongly disagree) to 5 (strongly agree). The total score ranges from 29 to 145, with higher scores indicating greater knowledge. The scale does not have a cut-off point. Eleven items (5, 8, 10, 12, 13, 14, 15, 16, 17, 21, 28) are reverse scored. The internal consistency of the instrument, as measured by Cronbach’s alpha, was found to be 0.86.

#### Pediatric nurses’ self-efficacy scale for pain management in children

This scale was developed by Bektaş, et al. [18] to determine pediatric nurses’ self-efficacy for pediatric pain management. Scale development studies were carried out between October 2020 and January 2021. Scale’s items include physiological, behavioral, and cognitive effects of pain in children, measurement tools used for pain assessment, pharmacological and non-pharmacological methods used in pediatric pain management, pain management in nonverbal children (e.g., ventilated or disabled children), and decision-making skills of nurses. The scale consists of 26 items and three sub-dimensions: The first sub-dimension, “Knowledge and Assessment,” comprises 11 items; the second sub-dimension, “Decision Making,” includes eight items; and the third sub-dimension, “Planning and Management,” consists of 7 items. The scale is rated on a 5-point Likert-type (1 = Absolutely not competent, 2 = Slightly competent sufficient, 3 = Moderately competent, 4 = Competent, 5 = Absolutely competent. The total score ranges from 26 to 130, with higher scores indicating greater self-efficacy in pediatric pain management. The scale has no cut-off point. The scale does not have any reverse-scored items. The Cronbach’s α value of the scale is 0.96.

Researchers administered data collection forms face-to-face during the Fall and Spring semesters of the 2021–2022 academic year. Completing the forms took approximately 20 min for each student. Students who did not agree to participate and wanted to leave the study at any stage were excluded. The study’s independent variables consisted of demographic data, while the dependent variables were pain management knowledge and self-efficacy levels.

### Data analysis

All statistical procedures were performed using SPSS version 24.0 software. Descriptive data were analyzed using frequencies, percentages, and means. The normality of scale scores was assessed using skewness and kurtosis values. The relationship between scale sub-dimensions was examined with correlation analysis. The predictive level of Nursing Students’ Pediatric Pain Management Knowledge Scale sub-dimensions on Pediatric Pain Self-Efficacy Scale sub-dimensions was evaluated with linear regression analysis. Variance Inflation Factor (VIF) and tolerance metrics were applied to detect multicollinearity among predictors. The significance level was considered as 0.05.

### Ethical consideration

The study complied with the ethical standards outlined in the Declaration of Helsinki. Ethical clearance was granted by the Non-Interventional Research Ethics Committee of a University (Date: 29.11.2021, No: 61351342/KASIM 2021-62) and the Nursing Department administration of the University. Participants were informed about the study objectives, and written informed consent was obtained prior to data collection. Following this, the forms were administered face-to-face. Permission to use the scale was also obtained from the scale authors via email.

## Results

The mean age of the students was 21.40 ± 1.38 years. Among the participants, 59% were female, 51.1% had received education on pain, and 89.3% had obtained it through their courses. Additionally, 57.3% of the students perceived themselves as moderately competent in pain management.

**Table 1:** Prediction of nursing students’ pediatric pain management knowledge scores on the “Knowledge and Assessment” Sub-Dimension of the pediatric pain management Self-Efficacy scale

Sub-Dimensions of Pediatric Pain Management Knowledge	Knowledge and Assessment				
	B	SE	β	t	*p*
**(constant)**	29.100	4.111		7.079	0.000
Pain Awareness	0.200	0.234	0.104	0.855	0.394
Pathophysiology	− 0.062	0.060	− 0.039	-1.028	0.305
Barriers	0.555	0.025	0.798	21.992	0.000
Diagnosis	0.317	0.074	0.124	4.297	0.000
Assessment	− 0.168	0.420	− 0.038	− 0.400	0.690
Pain Control	0.065	0.243	0.026	0.267	0.790
**R**	0.219				
**R** ^**2**^	0.048				
**F**	1.680				
**P**	0.127				
DW** (1,**5 − 2**,**5)**	1.574				

** DW: Durbin Watson

The sub-dimensions of pediatric pain management knowledge did not significantly predict the “Knowledge and Assessment” sub-dimension of the Pediatric Pain Management Self-Efficacy Scale (*p* > 0.05, Table 1).

**Table 2:** Prediction of nursing students’ pediatric pain management knowledge scores on the “Decision Making” Sub-Dimension of the pediatric pain management Self-Efficacy scale

Sub-Dimensions of Pediatric Pain Management Knowledge	Decision Making				
	B	SH	β	t	*p*
**(constant)**	1.538	0.883		1.743	0.083
Pain Awareness	0.080	0.050	0.070	1.607	0.110
Pathophysiology	− 0.062	0.060	− 0.039	-1.028	0.305
Barriers	0.555	0.025	0.798	21.992	0.000
Diagnosis	0.317	0.074	0.124	4.297	0.000
Assessment	0.299	0.088	0.117	3.397	0.001
Pain Control	0.008	0.052	0.005	0.148	0.882
**R**	0.934				
**R** ^**2**^	0.874				
**F**	234.047				
**P**	0.000				
DW** (1,**5 − 2**,**5)**	1.961				

** DW: Durbin Watson

The sub-dimensions of the Pediatric Pain Management Knowledge Scale significantly predicted the “Decision Making” sub-dimension of the Pediatric Pain Management Self-Efficacy Scale (*p* < 0.01, Table 2). The pediatric pain knowledge scale sub-dimensions explained 87.4% of the variance in the pediatric pain self-efficacy scale “Decision Making” sub-dimension score. When examining which variables significantly predicted the “Decision Making” sub-dimension individually, it was found that the knowledge levels related to “barriers on pain,” “pain diagnosis,” and “pain assessment” significantly contributed to the prediction (*p* < 0.05).

**Table 3:** Prediction of nursing students’ pediatric pain management knowledge scores on the “Planning and Management” Sub-Dimension of the pediatric pain management Self-Efficacy Scale

Sub-Dimensions of Pediatric Pain Management Knowledge	Planning and Management				
	B	SH	β	t	*p*
**(constant)**	11.554	2.888		4.000	0.000
Pain Awareness	0.214	0.163	0.149	1.310	0.192
Pathophysiology	0.637	0.196	0.317	3.247	0.001
Barriers	− 0.061	0.082	− 0.070	− 0.740	0.460
Diagnosis	0.163	0.240	0.051	0.679	0.498
Assessment	− 0.318	0.290	− 0.098	-1.098	0.274
Pain Control	0.047	0.171	0.025	0.277	0.782
**R**	0.384				
**R** ^**2**^	0.148				
**F**	5.854				
**P**	0.000				
DW** (1,**5 − 2**,**5)**	1.756				

** DW: Durbin Watson

The analysis revealed that the sub-dimensions of the Pediatric Pain Management Knowledge Scale significantly predicted the “Planning and Management” sub-dimension of the Pediatric Pain Management Self-Efficacy Scale (*p* < 0.01, Table 3). The Pediatric Pain Management Knowledge Scale sub-dimensions explained 14.8% of the variance in the “Planning and Management” sub-dimension of the Pediatric Pain Management Self-Efficacy Scale. When examining which variables significantly contributed to this prediction, it was found that only “Pain Pathophysiology” knowledge was a significant individual predictor of the “Planning and Management” sub-dimension (*p* = 0.01).

**Table 4:** The relationship Between Sub-Dimensions of pediatric pain management knowledge scale and Sub-Dimensions of pediatric pain management Self-Efficacy scale of nursing students

Variables	Knowledge and Assessmentr	Decision Making *r*	Planning and Management *r*
Pain Awareness	0.115	0.682^**^	0.295^**^
Pathophysiology	0.112	0.598^**^	0.328^**^
Barriers	− 0.011	0.912^**^	0.175^**^
Diagnosis	0.135^*^	0.320^**^	0.197^**^
Assessment	0.033	0.641^**^	0.131
Pain Control	0.089	0.481^**^	0.219^**^

* Correlation is significant at the 0.05 0.05 level (two-tailed); ** Correlation is statistically significant at the 0.01 level (two-tailed)

Total scores on the pediatric pain knowledge scale were not significantly correlated with self-efficacy levels (*p* > 0.05). However, a strong and significant correlation was identified between the students’ “Pain Awareness” subdimension score of the Pediatric Pain Management Knowledge Scale and the “Decision-Making” subdimension of the Pediatric Pain Management Self-Efficacy Scale, while a weak significant correlation was found with the “Planning and Management” subdimension (*p* < 0.05). Similarly, a strong and significant correlation was found between the students’ “Pain Pathophysiology” subdimension score of the Pediatric Pain Management Knowledge Scale and the “Decision-Making” subdimension of the Pediatric Pain Management Self-Efficacy Scale, whereas a weak but significant correlation was found with the “Planning and Management” subdimension (*p* < 0.05). Furthermore, a very strong and significant correlation was detected between the students’ “Barriers” subdimension score and the “Decision-Making” subdimension, along with a weak but significant correlation with the “Planning and Management” subdimension (*p* < 0.05). A weak yet significant correlation was noted between the students’ “Pain Diagnosis” subdimension score and all three subdimensions of the Pediatric Pain Management Self-Efficacy Scale: “Knowledge and Evaluation,” “Decision-Making,” and “Planning and Management” (p < 0.05). Additionally, a strong and significant correlation was found between the “Pain Assessment” subdimension and the “Decision Making” subdimension scores (p < 0.05). Finally, a significant and strong association was found between knowledge of “Pain Control” and self-efficacy in “Decision Making”, while a weak but significant correlation was found with the “Planning and Management” subdimension (p < 0.05, Table 4).

## Discussion

This study aims to determine to what extent knowledge of pediatric pain management predicts nursing students’ self-efficacy. A literature review revealed a limited number of studies on this issue. Therefore, the findings of this study were discussed in relation to existing research that assessed pediatric pain management knowledge, attitudes, beliefs, and self-efficacy levels among nurses and nursing students using various measurement tools.

Nurses’ knowledge levels regarding pediatric pain management can influence their self-efficacy [19]. Findings from this research indicate that students’ knowledge alone did not serve as a predictor of their self-efficacy in managing pediatric pain. However, a study conducted by Belli [13] found a significant relationship between pediatric pain management knowledge levels and self-efficacy of students taking a pediatric nursing course. The discrepancy between the current study’s findings and Belli’s (2020) research may be attributed to differences in the characteristics of the sample groups. Notably, the proportion of students who had received pain-related education in this study (51.1%) was considerably higher than that reported in Belli’s (2020) study (3.3%).

Educational initiatives in pain management are critical, as knowledge deficits among pediatric nurses have been linked to compromised care quality and lower patient satisfaction levels [20]. Nonetheless, when self-efficacy is not supported by sufficient knowledge, it may lead to suboptimal practices in pediatric pain care. Indeed, a study conducted by Parvizy, Tarvirdinasab [21] examined the effects of pain management education on the knowledge, attitudes, and self-efficacy of pediatric nurses, revealing that both knowledge and self-efficacy levels significantly increased in the intervention group that received training. This indicates a potential mismatch between perceived competence and actual knowledge levels among nurses in pain management. Such a gap could pose a barrier to advancing the pain management competencies of the broader healthcare team. In the present study, the participants’ pediatric pain management knowledge scores did not predict their self-efficacy in “Knowledge and Assessment” because the students were still in the education phase and their theoretical knowledge had not yet been reinforced through clinical practice. In alignment with our findings, Ha and An [14] reported inadequate pediatric pain knowledge among undergraduate nursing students. However, significant differences in self-efficacy were observed among the group with above-average knowledge scores.

It was determined that the sub-dimensions of the pediatric pain management knowledge level of the students participating in this study did not significantly predict the scores of the “Knowledge and Assessment” sub-dimensions of the Pediatric Pain Management Self-Efficacy Scale (*p* > 0.05). Supporting this outcome, Stanley and Pollard [22] also observed no significant correlation between pediatric nurses’ pain knowledge and their self-efficacy (*r* = 0.039, *p* = 0.853). In that study, despite nurses reporting high levels of self-efficacy, their pain management knowledge scores were found to be low. In the present study, when the student nurses were asked about their perceived competence in pediatric pain management, 57.3% stated they felt “partially competent.” This finding may be explained by the fact that the students have not yet completed their professional education and lack sufficient clinical experience, leading to a lower sense of competence.

In the present study, the knowledge of nursing students about subdimensions of “Barriers,” “Pain Diagnosis,” and “Pain Assessment” significantly predicted “Decision-Making” self-efficacy. Knowledge-related factors accounted for 87.4% of the variance observed in students’ decision-making self-efficacy scores. Heo, Kim [23] investigated barriers in pediatric pain practices and identified child- and parent-related factors as key obstacles. Notably, nurses were found to have low self-efficacy in assessing pain according to children’s developmental stages. Additionally, the study revealed that nurses had relatively low utilization rates of pain assessment and non-pharmacological interventions within pain management practices. The key factors significantly affecting pediatric pain management practices were division of labor and self-efficacy. It was determined that these two factors explained 37.5% of the variance in pain management practices. The findings between these studies may be attributed to differences in sample groups. The present study sample consisted of nursing students without sufficient clinical experience or independent decision-making competence. This might clarify why decision-making self-efficacy was notably influenced by their understanding of barriers, diagnosis, and assessment domains. The high level of theoretical knowledge acquired during their ongoing nursing education may have contributed to their self-efficacy.

Additionally, support from clinical mentors or faculty members in problem-solving during clinical practice may have further enhanced their self-efficacy. However, in Heo et al. (2016) study, which focused on pediatric nurses, low self-efficacy in addressing pain management barriers, age-specific pain assessment, and the use of non-pharmacological pain management methods could be explained by their insufficient knowledge of pain management and the necessity to confront these challenges firsthand in clinical settings, making individual decisions in real-world scenarios. This aligns with the finding that the average correct response rate in the Pediatric Pain Management Knowledge Scale was only 58.8%.

This study determined that nursing students’ knowledge of pain pathophysiology significantly predicted their self-efficacy in “Planning and Management.” This outcome may reflect the foundational pain-related education offered throughout the nursing curriculum. The emphasis on pain pathophysiology in coursework contributed to students’ higher self-efficacy in planning care and effectively managing pain in clinical settings.

A well-informed and skillful healthcare team is essential for achieving optimal results in pain control efforts [1]. Studies in the literature indicate that nurses’ knowledge, attitudes, and self-efficacy in pain management are insufficient and should be improved or reconsidered through education [21, 24, 25]. In the present study, a strong correlation was found between nursing students’ knowledge levels in “Pain Awareness,” “Pathophysiology,” “Barriers,” “Pain Assessment,” and “Pain Control” and their “Decision-Making” self-efficacy. Similarly, a study conducted by Sayin Kasar and Kutmec Yilmaz [26] reported that as nursing students’ confidence in their self-efficacy in pain management increased, their attitudes toward patients experiencing pain also became more positive. The results suggest a direct connection between students’ pain knowledge and both their confidence in clinical decisions and their outlook toward managing patient discomfort. Therefore, enhancing structured curricula and practical training regarding pain management within nursing education is crucial to strengthening students’ self-efficacy and clinical decision-making skills.

### Implications for nursing practice

The findings of this study underscore the necessity of integrating comprehensive pediatric pain management training into undergraduate nursing education. Although students’ general knowledge of pediatric pain management did not significantly predict their overall self-efficacy, specific knowledge areas—particularly those related to pain barriers, diagnosis, and assessment—were found to strongly influence decision-making self-efficacy. This highlights the importance of focusing not only on foundational knowledge, but also on skill-based and case-oriented training that improves students’ confidence in making clinical decisions. Therefore, nursing curricula should be revised to include simulation-based scenarios and structured clinical mentoring programs that focus on identifying and overcoming pain management barriers, conducting accurate assessments, and planning evidence-based interventions for pediatric patients.

Furthermore, the significant association between knowledge of pain pathophysiology and the “Planning and Management” subdimension of self-efficacy suggests that reinforcing pathophysiological concepts in clinical contexts can improve students’ ability to implement effective care plans. Educational strategies should promote the development of cognitive frameworks that support critical thinking and clinical reasoning in pain management. Interdisciplinary collaborations, case reflections, and guided clinical debriefings may serve as valuable tools for translating theoretical knowledge into practice. By adopting a multifaceted educational approach that nurtures both knowledge and self-efficacy, nurse educators can enhance students’ readiness to deliver competent and empathetic care to pediatric patients experiencing pain.

### Limitations

This study has several limitations. First, it was conducted with nursing students from a single state university in Türkiye, which may limit the generalizability of the findings to other institutions or educational contexts. Second, the cross-sectional design does not allow for causal inferences between knowledge and self-efficacy. Third, the data were collected through self-report instruments, which may be subject to social desirability bias or inaccuracies in participants’ perceptions. Additionally, while the study identified significant relationships between knowledge subdimensions and self-efficacy, other factors such as clinical experience, teaching methods, and individual motivation were not assessed and could have influenced the outcomes. Future studies using longitudinal designs and broader, more diverse samples are recommended to confirm and expand upon these findings.

## Conclusions

While nursing students’ overall knowledge did not significantly predict their self-efficacy in managing pediatric pain, the study revealed strong and meaningful correlations between specific knowledge areas—such as pain awareness, pathophysiology, perceived barriers, assessment, and control—and their confidence in making clinical decisions. These results underscore the nuanced relationship between knowledge and confidence in clinical practice. The findings emphasize the need for nursing curricula to go beyond theoretical instruction and focus on enhancing students’ practical decision-making skills in pediatric pain management. Incorporating targeted education on pain assessment, recognition of care barriers, and strategy development may contribute to more competent and self-assured nursing professionals, thereby improving care outcomes for pediatric patients.

Future research should examine the long-term effects of educational interventions on nursing students’ self-efficacy and clinical competence in pain management. Longitudinal and experimental designs could offer deeper insight into how specific knowledge domains translate into confident, evidence-based practice. Exploring the role of simulation-based training and clinical mentoring may also provide valuable contributions to this area.

## Data Availability

The data that support the findings of this study are available from the corresponding author upon reasonable request.

## Abbreviations

SPSS: Statistical Package for Social Sciences
VIF: Variance Inflation Factor
DW: Durbin Watson
